# Visual motion sensitivity as an indicator of diabetic retinopathy in type 2 diabetes mellitus

**DOI:** 10.3389/fnins.2024.1412241

**Published:** 2024-08-02

**Authors:** Tianlin Zhang, Haojiang Ying, Huiqun Wang, Fouxi Zhao, Qiying Pan, Qingqing Zhan, Fuyan Zhang, Qinyu An, Tao Liu, Yuandong Hu, Yang Zhang

**Affiliations:** ^1^School of Public Health, The Key Laboratory of Environmental Pollution Monitoring and Disease Control, Ministry of Education, Guizhou Medical University, Guiyang, China; ^2^Department of Psychology, Soochow University, Suzhou, China; ^3^Guizhou Center for Disease Control and Prevention, Guiyang, China; ^4^Medical College, Guizhou University, Guiyang, China

**Keywords:** visual motion sensitivity, second-order, diabetic retinopathy, type 2 diabetes mellitus, cross-sectional study

## Abstract

**Objectives:**

This current study is based on a set of visual motion sensitivity tests, investigating the correlation between visual motion sensitivity and diabetic retinopathy (DR) in type 2 diabetes mellitus (T2DM), thereby furnishing a scientific rationale for preventing and controlling DR.

**Methods:**

This research was conducted by a combination of questionnaire collection and on-site investigation that involved 542 T2DM recruited from a community. The visual motion sensitivity determined the visual motion perception of the participants across three spatial frequencies (low, medium, and high) for both the first- and second-order contrast. The logistic regression model was adopted to investigate the relationship between visual motion sensitivity and DR prevalence. Besides, the Pearson correlation analysis was used to analyze the factors influencing visual motion sensitivity and restricted cubic spline (RCS) functions to assess the dose–response relationship between visual motion sensitivity and glycated hemoglobin.

**Results:**

Among 542 subjects, there are 162 cases of DR, with a prevalence rate of 29.89%. After adjusting factors of age, gender, glycated hemoglobin, duration of diabetes, BMI, and hypertension, we found that the decline in first- and second-order high spatial frequency sensitivity increased the risk for DR [odds ratio (OR): 1.519 (1.065, 2.168), 1.249 (1.068, 1.460)]. The decline in perceptual ability of second-order low, medium, and high spatial frequency sensitivity is a risk factor for moderate to severe DR [OR: 1.556 (1.116, 2.168), 1.388 (1.066, 1.806), 1.476 (1.139, 1.912)]. The first-order and the second-order high spatial frequency sensitivity are significantly positively correlated with glycated hemoglobin (*r* = 0.105, *p* = 0.015 and *r* = 0.119, *p* = 0.005, respectively).

**Conclusion:**

Visual motion sensitivity especially for the second-order high spatial frequency stimuli emerges as a significant predictor of DR in T2DM, offering a sensitive diagnostic tool for early detection.

## 1 Introduction

Diabetes mellitus is a prevalent metabolic disorder that arises from various pathogenic factors that impair pancreatic function or insulin resistance, with hyperglycemia as its main clinical feature (Deshpande et al., 2008). Predominantly, more than 90% of cases of diabetes are categorized as type 2 diabetes mellitus (T2DM) (Sun et al., 2022). Uncontrolled diabetes can precipitate systemic damage across various tissues and organs, with notably severe impacts on the eye, cardiovascular, cerebrovascular, and nervous systems, diabetic retinopathy (DR) is the primary ocular damage in patients with T2DM (Karoli et al., 2013). Due to its significant impact on visual function, Diabetic Retinopathy (DR) is a critical concern and is considered one of the primary causes of visual impairment and blindness among adults worldwide (Teo et al., 2021). Research indicates that the prevalence of DR in type 2 diabetes in China is 30.1% (Li et al., 2020). The pathophysiology of DR encompasses blood-retinal barrier, retinal neovascularization, and macular edema, ultimately leading to potential blindness (Heng et al., 2013). Due to the compensatory and plastic nature of the human visual nervous system, minor organic lesions may not necessarily result in subjectively observable visual perceptual impairments (Horton et al., 2017). Therefore, in the initial stages of DR development, patients may not exhibit noticeable symptoms, leading to a missed opportunity for early intervention. Consequently, it is imperative to conduct regular screenings for this disease to facilitate prompt treatment of retinal lesions and prevent vision loss.

Research has shown that close follow-up and early intervention at earlier stages are important for the prevention of disease progression of other diabetes mellitus complications (Lee et al., 2021). However, the screening for DR is frequently hindered by inadequate accessibility and dependence on clinical examination and fundus imaging. Therefore, it is imperative to improve cost-effective DR screening programs and increase the accessibility of DR screening. Furthermore, previous research has suggested that persistent hyperglycemia in individuals with diabetes may negatively impact cognitive function, which could result in cognitive impairment in type 2 diabetes mellitus (Cosway et al., 2001; Asimakopoulou et al., 2002). Thus, identifying sensitive indicators for assessing visual function could offer early intervention opportunities for DR and may even serve as an early screening tool for other vascular variations caused by diabetes, such as organic brain lesions.

Visual impairment is an external manifestation of DR, thus visual function tests that align with the pathological nature can be regarded as a starting point. Visual motion sensitivity is a significant indicator of cognitive function in the visual cortex, which reflects the degree of change in the visual system’s cognitive ability toward moving objects and has critical biological significance (Derrington et al., 2004). This study distinguishes between two types of visual motion sensitivity: first-order and second-order spatial frequency sensitivity. First-order spatial frequency sensitivity is based on changes in brightness, while second-order spatial frequency sensitivity is based on changes in texture contrast. The findings of this study suggest a clear division between these two types of sensitivity. The first- and second-order spatial frequency sensitivity depends on different information processing mechanisms and neural systems (Lu and Sperling, 2001). Vaina studied multiple brain-damaged patients and found that in patients with unilateral brain damage, second-order motion perception was impaired while first-order motion perception remained intact, indicating the existence of different pathways for motion perception (Vaina and Cowey, 1996). Ledgeway et al.’s research findings suggest the existence of separate horizontal motion mechanisms for first-order and second-order motion, with each mechanism being insensitive to stimuli of the other type (Ledgeway and Smith, 1994). In addition, the psychophysical evidence for the view that independent pathways process first- and second-order motion stimuli includes: differential temporal sensitivity to first-order and second-order motion stimuli (Glasser and Tadin, 2011), first-order rather than second-order motion perception at absolute detection thresholds (Smith and Ledgeway, 1997), and a small phase dependence in direction discrimination experiments with superimposed Fourier and non-Fourier stimuli (Lu and Sperling, 1995). The aforementioned studies suggest that the human visual system possesses distinct and specialized mechanisms for detecting first- and second-order motion. Consequently, it can be posited that individuals with varying degrees of visual impairment may exhibit varying degrees of impairment in their first-order versus second-order visuomotor cognitive abilities. As such, the degree of visual impairment can be inferred inversely from the observed deterioration processes. If visual acuity impairment occurs during the early stages of DR, a screening method for DR could be established through visual acuity testing, thus providing a convenient and cost-effective means for DR screening. This would have implications for both preventing and controlling the disease.

By examining the relationship between visual motion sensitivity across different spatial frequencies (low, medium, and high) and DR in patients with T2DM, the current study seeks to investigate the effects of first- and second-order spatial frequency stimulus on the prevalence of DR in type 2 diabetes patients, thereby exploring the predictive value of visual acuity for early-onset DR.

## 2 Materials and methods

### 2.1 Recruitment and study population

This study recruited 2 type 2 diabetes patients from four communities in Guizhou Province between February and September 2022, excluding those with a history of mental illness, eye surgery, or any condition that would affect fundus examination. A total of 687 cases of type 2 diabetes were recruited, with 615 cases completing the examination; inclusion and exclusion criteria were used for screening to ensure that the analysis data was complete, and a total of 542 cases met the conditions for this study. All study subjects signed informed consent forms. This study was approved by the Ethics Committee of the Guizhou Center for Disease Control and Prevention (S2023-12), and all subjects signed informed consent.

### 2.2 On-site investigation

#### 2.2.1 Questionnaire survey

A questionnaire was administered to the respondents, which was self-designed. The questionnaire includes age, gender (male, female), hypertensive condition (whether diagnosed with hypertension), duration of diabetes, etc. The questionnaire survey was conducted by trained investigators in face-to-face interviews.

#### 2.2.2 Glycated hemoglobin test

Measurement of glycated hemoglobin fractions using the Sinocare (PCH-100) Portable Glycated Hemoglobin Analyzer (PCH-100HbA1c kit).

#### 2.2.3 Blood pressure detection

Blood pressure values were measured using a Yuwell (YE660A) electronic sphygmomanometer, hypertension is defined as: a self-reported diagnosis of hypertension by a physician or antihypertensive treatment, or systolic blood pressure ≥ 140 mmHg (18.6 kPa) and/or diastolic blood pressure ≥ 90 mmHg (12.0 kPa).

#### 2.2.4 Physical examination

Height and weight measurements were obtained using the Shanghe (SH-20A) measuring instrument. Body Mass Index (BMI) was calculated as weight (kg) divided by height squared (m^2^). Visual acuity was assessed using a standard logarithmic chart. The 5SL chart was printed on offset paper, with a brightness of 200 cd/m^2^ and dimensions of 777 × 217 millimeters. The chart was positioned 5 meters in front of the subject’s eyes. The minimum vision that the subject could recognize was determined when the number of correctly identified letters exceeded half of the total number of letters in the line. Visual acuity was measured using the 5-point recording system within the range of 4.0–5.3. In cases where there was inconsistency in the vision results between both eyes, the final diagnosis was based on the eye with better vision.

#### 2.2.5 Routine examination of DR: fundus imaging

All participants were brought into a completely dark room and the MOCULAR (ML-800 s) fundus camera was used for retinal imaging photography. Each subject has a color photo of the macula centered for both the left and right eyes. Cases where fundus imaging could not be staged due to poor image quality or opacity were excluded from the analysis. Fundus imaging was taken by trained staff to ensure the quality of the images; followed by rigorous diagnosis by ophthalmologists based on the latest Chinese edition of the clinical guidelines for diagnosing and treating DR, ensuring the quality of the diagnostic stages for DR. Six ophthalmic experts diagnosed and staged the fundus photographs of the subjects based on the Chinese Clinical Guidelines for Diabetic Retinopathy (Xun and Xiaoxin, 2023). The specific staging criteria are: (1) Mild non-proliferative DR (NPDR): only capillary tumor-like swelling changed; (2) Moderate NPDR: may be associated with retinal bleeding, rigid exudation, and cotton freckles; (3) Severe NPDR: ≥20 bleeding points per quadrant of retinal bleeding, or at least 2 quadrants of defined venous “bead-like” changes, or at least 1 quadrant of retinal microvascular abnormalities; (4) proliferative DR(PDR): retinal neovascularization or disc neovascularization occurred; there are fibrous vascular membranes, which can be accompanied by anterior retinal hemorrhage or vitreous volume blood; traction retinal detachment, which can be combined with fibrous blood vessel, retinal blood accumulation or vitreous blood volume. If the photograph of one eye is indiscernible, the final diagnosis will be determined based on the available photograph; when the staging results of both eyes are inconsistent, the final diagnosis is determined by the eye with the worst staging result.

#### 2.2.6 Visual motion sensitivity test

##### 2.2.6.1 Testing equipment

Using the XGIMI (RSPro2) projector, with a resolution of 3,840 pixels x 2,160 pixels, the projected screen size is 1.6 meters x 0.9 meters.

##### 2.2.6.2 Testing materials

**Figure 1:**
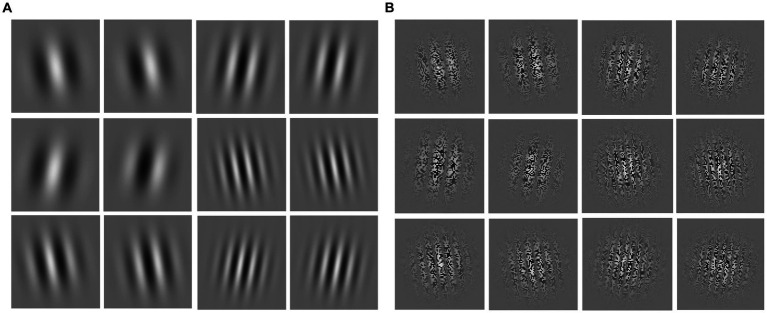
Screenshots of first- and second-order spatial frequency stimulus materials. **(A)** First-order spatial frequency stimulus material; **(B)** second-order spatial frequency stimulus material; from top to bottom are low, medium, and high spatial frequency stimuli, respectively.

The visual motion sensitivity test material consists of 24 videos, each presenting a “first-order” or “second-order” spatial frequency stimulus material (50% each). First-order spatial frequency stimuli are simple unit shifts based on luminance changes, while second-order motion spatial frequency stimuli are visual stimuli based on contrast changes. First- and second-order spatial frequency stimuli were categorized as low spatial frequency motion, medium spatial frequency motion, and high spatial frequency motion (33.3% of each) based on the different spatial frequencies of the motion visual stimuli. In Figures 1A,B respectively depict screenshots of first- and second-order spatial frequency stimulus. Each row of four images represents a screenshot of a frequency motion, from top to bottom illustrating low, medium, and high spatial frequency sensitivity. The variations in stimuli cycles for low, medium, and high spatial frequencies are 4, 7, and 10 cycles; the stimulus frequencies are 4, 7, and 10 cycles/image, with spatial frequencies of 0.089, 0.208, and 0.2967 cycles per degree, and the viewing angles for all 24 test materials were measured at 82.19 degrees.

##### 2.2.6.3 Testing process

The test is performed in a quiet room that is sheltered from light. In the process of detection, 24 test videos were randomly played to prevent subjects from discovering test rules. Each test material lasts for 9 s, with the first 4 s serving as a countdown to allow participants to acclimate. The specific visual motion sensitivity test consists of 1 s, after which a blank screen appears upon completion of the test content. The remaining 4 s of blank space are used by the participants to make their judgments. Upon recording the participant’s judgment, the tester immediately proceeds to play the next testing material. The subjects sit in front of the projection screen at a distance of 1.2 meters, with their eyes perpendicular to the central position of the screen. They determine the directional movement of patterns in 24 videos (from left to right or from right to left) and subsequently communicate their judgments to the testers.

##### 2.2.6.4 Interpretation of test results

The testers determine if the direction (“left” or “right”) of observed motion stimuli by the subjects is incorrect. If so, it is scored as 1 point, with higher scores indicating lower visual motion sensitivity.

### 2.3 Statistical analysis

The continuous variables are described as “mean ± standard deviation,” and the comparison between different groups is conducted using t-tests. Categorical variables were described as frequency (percentage) and compared by the chi-square test. The difference between the sample of this study and the conventional sample in China was compared using the two-sample test with a normal approximation of the binomial distribution. The association between visual motion sensitivity and the risk of DR was estimated by the Logistic regression model. The respective associations are determined by the odds ratio (OR) and their 95% confidence intervals (95% CI). A value greater than 1 indicates a positive correlation, while a value less than 1 indicates a negative correlation. There are a total of three Models: Model I only incorporates visual motion sensitivity, focusing on the impact of visual-motor sensitivity on DR; Model II builds upon Model I by incorporating age and gender adjustments, eliminating the significant demographic factors that cannot be changed to study the relationship between visual-motor sensitivity and DR; Model III incorporated factors such as glycated hemoglobin, duration of diabetes, body mass index (BMI), and hypertension (all known risk factors for diabetic retinopathy), thereby enhancing the credibility of visual acuity about diabetic retinopathy. Exploring the correlation between visual motion sensitivity and other factors using Pearson correlation analysis, a correlation coefficient (*r*) greater than 0 indicates a positive correlation, whereas a value less than 0 indicates a negative correlation, and subgroup analysis by gender (male; female), age (age<65 groups; age ≥ 65 groups), and diabetes duration (grouped by the median of diabetes duration: diabetes duration<8 groups; diabetes duration≥8 groups). The dose–response relationship analysis will be conducted using restricted cubic splines.

Data analysis was performed using SPSS version 26.0. Restricted cubic spline plots were generated using R version 4.2.2. All statistical tests were two-sided and *p* < 0.05 was considered statistically significant.

## 3 Results

### 3.1 Demographic and clinical characteristics of the study participants

Among 542 patients with type 2 diabetes, 162 were diagnosed with DR, resulting in a prevalence rate of 29.89%. Research indicates that the prevalence of DR in type 2 diabetes in China is 30.1% (Li et al., 2020). According to the normal approximation method of the binomial distribution, the difference in the prevalence of DR between this study and that in China is not statistically significant (*u* = −0.075, *p* = 0.299).

The prevalence of DR is 30.33% among males and 29.53% among females, with no statistically significant difference in the prevalence between genders (*p* = 0.840). Categorical variables such as gender and hypertension were analyzed using chi-square tests, while continuous variables such as age and BMI were analyzed using *t*-tests. The results showed that there is no statistically significant difference in vision (measured by a logarithmic visual acuity chart) between the DR group and non-DR group (*p* = 0.602). Compared with the non-DR group, the DR group has significantly higher levels of diabetes duration (*p* = 0.029), glycated hemoglobin (*p* = 0.001), visual motion sensitivity (*p* = 0.002), first-order high spatial frequency sensitivity (*p* = 0.009), second-order high spatial frequency sensitivity (*p* = 0.001) (Table 1).

**Table 1:** The distribution pattern of DR among general characteristics.

Characteristics	Total (*n* = 542)	Non-DR (*n* = 380)	DR (*n* = 162)	*t*/χ^2^	*p*
Sex					
Male	244	170 (69.67)	74 (30.33)	0.041	0.840
Female	298	210 (70.47)	88 (29.53)		
Hypertension					
Yes	245	177 (72.24)	68 (27.76)	−0.972	0.324
No	297	203 (68.35)	94 (31.65)		
Age	66.19 ± 8.91	66.23 ± 8.86	66.08 ± 9.05	−0.181	0.271
BMI	24.63 ± 3.22	24.71 ± 3.29	24.44 ± 3.06	−0.896	0.371
Vision	4.72 ± 0.24	4.73 ± 0.23	4.7 ± 0.26	−1.101	0.602
Diabetes duration	9.14 ± 7.28	8.69 ± 7.27	10.19 ± 7.23	2.193	0.029
Glycated hemoglobin	7.22 ± 2.26	7.00 ± 2.23	7.72 ± 2.23	3.437	0.001
Visual motion sensitivity	3.94 ± 3.15	3.66 ± 2.9	4.57 ± 3.6	3.106	0.002
First-order low spatial frequency sensitivity	0.13 ± 0.41	0.11 ± 0.37	0.17 ± 0.49	1.622	0.105
First-order medium spatial frequency sensitivity	0.15 ± 0.44	0.13 ± 0.4	0.19 ± 0.51	1.587	0.113
First-order high spatial frequency sensitivity	0.16 ± 0.50	0.12 ± 0.46	0.25 ± 0.59	2.624	0.009
Second-order low spatial frequency sensitivity	0.77 ± 0.95	0.73 ± 0.89	0.87 ± 1.09	1.555	0.133
Second-order medium spatial frequency sensitivity	1.26 ± 1.15	1.21 ± 1.09	1.37 ± 1.28	0.970	0.332
Second-order high spatial frequency sensitivity	1.47 ± 1.20	1.36 ± 1.13	1.72 ± 1.32	3.216	0.001

### 3.2 Analysis of the relationship between visual motion sensitivity and DR

This analysis uses logistic regression to study the relationship between visual motion sensitivity and DR (*n* = 162). The independent variable is visual motion sensitivity (first- and second-order spatial frequency sensitivity), and the dependent variable is DR (with the non-DR group as the reference). The results show that after adjusted for age and gender, the first-order high spatial frequency sensitivity [*p* = 0.009, OR (95% CI):1.588 (1.122, 2.247)] and second-order high spatial frequency sensitivity [*p* = 0.001, OR (95% CI):1.283 (1.100, 1.496)] were significantly associated with increased risks of DR. After further adjusted for glycated hemoglobin, diabetes duration, BMI, and hypertension, first-order high spatial frequency sensitivity [*p* = 0.021, OR (95% CI):1.519 (1.065, 2.168)], and second-order high spatial frequency sensitivity [*p* = 0.005, OR (95% CI):11.249 (1.068, 1.460)] were still significantly associated with the risk of DR (Table 2).

**Table 2:** Logistic analysis of the relationship between visual motion sensitivity and DR.

Model	Visual motion sensitivity	*B*	*p*	OR (95%CI)
Model I	First-order low spatial frequency sensitivity	0.344	0.110	1.411 (0.926, 2.151)
	First-order medium spatial frequency sensitivity	0.317	0.117	1.373 (0.924, 2.040)
	First-order high spatial frequency sensitivity	0.442	0.011	1.556 (1.106, 2.189)
	Second-order low spatial frequency sensitivity	0.149	0.121	1.161 (0.961, 1.402)
	Second-order medium spatial frequency sensitivity	0.121	0.134	1.128 (0.964, 1.321)
	Second-order high spatial frequency sensitivity	0.247	0.002	1.281 (1.099, 1.493)
Model II	First-order low spatial frequency sensitivity	0.361	0.097	1.435 (0.936, 2.200)
	First-order medium spatial frequency sensitivity	0.334	0.103	1.397 (0.934, 2.089)
	First-order high spatial frequency sensitivity	0.462	0.009	1.588 (1.122, 2.247)
	Second-order low spatial frequency sensitivity	0.158	0.106	1.171 (0.967, 1.419)
	Second-order medium spatial frequency sensitivity	0.124	0.128	1.131 (0.965, 1.326)
	Second-order high spatial frequency sensitivity	0.249	0.001	1.283 (1.100, 1.496)
Model III	First-order low spatial frequency sensitivity	0.327	0.143	1.387 (0.895, 2.148)
	First-order medium spatial frequency sensitivity	0.347	0.100	1.415 (0.935, 2.140)
	First-order high spatial frequency sensitivity	0.418	0.021	1.519 (1.065, 2.168)
	Second-order low spatial frequency sensitivity	0.157	0.116	1.170 (0.962, 1.422)
	Second-order medium spatial frequency sensitivity	0.096	0.249	1.100 (0.935, 1.294)
	Second-order high spatial frequency sensitivity	0.222	0.005	1.249 (1.068, 1.460)

Model I, No adjustments were made; Model II, adjusts for age and gender; Model III, adjusts for age, gender, glycated hemoglobin, diabetes duration, BMI, and hypertension. Odds Ratio (OR); 95% confidence interval (95% CI); regression coefficient (B).

### 3.3 Analysis of the relationship between visual motion sensitivity and non-mild NPDR

The purpose of this section is to investigate the impact of first- and second-order spatial frequency sensitivity on the severity of DR lesions. Among 162 DR patients, there were 91 cases of mild NPDR, 54 cases of moderate NPDR, 10 cases of severe NPDR, and 7 cases of PDR. To ensure a balanced sample size for analysis, we divided DR into two groups: one group is mild NPDR (*n* = 91), and the other group is non-mild NPDR (including moderate-to-severe NPDR and PDR, *n* = 71). Logistic regression analysis was used with the independent variable being visual Motion Sensitivity (first-order spatial frequency sensitivity and second-order spatial frequency sensitivity), and the dependent variable being non-mild NPDR.

The results show that after adjusting for age, gender, glycated hemoglobin, diabetes duration, BMI, and hypertension, the second-order low spatial frequency sensitivity [*p* = 0.009, OR (95% CI):1.556 (1.116, 2.168)], second-order medium spatial frequency sensitivity[*p* = 0.015, OR (95% CI):1.388 (1.066, 1.806)], second-order high spatial frequency sensitivity[*p* = 0.003, OR (95% CI):1.476 (1.139, 1.912)] were significantly associated with increased risks of non-mild NPDR. However, first-order spatial frequency sensitivity was not significantly associated with increased risk of non-mild NPDR (Table 3).

**Table 3:** Logistic analysis of the relationship between visual motion sensitivity and non-mild NPDR.

Model	Visual motion sensitivity	*B*	*p*	OR (95%CI)
Model I	First-order low spatial frequency sensitivity	0.075	0.815	1.078 (0.575, 2.020)
	First-order medium spatial frequency sensitivity	0.440	0.175	1.552 (0.822, 2.930)
	First-order high spatial frequency sensitivity	0.249	0.355	1.283 (0.757, 2.177)
	Second-order low spatial frequency sensitivity	0.306	0.041	1.358 (1.013, 1.820)
	Second-order medium spatial frequency sensitivity	0.355	0.006	1.425 (1.108, 1.834)
	Second-order high spatial frequency sensitivity	0.382	0.003	1.465 (1.144, 1.877)
Model II	First-order low spatial frequency sensitivity	0.057	0.860	1.059 (0.560, 2.002)
	First-order medium spatial frequency sensitivity	0.448	0.179	1.566 (0.815, 3.010)
	First-order high spatial frequency sensitivity	0.271	0.323	1.311 (0.767, 2.242)
	Second-order low spatial frequency sensitivity	0.353	0.024	1.423 (1.048, 1.933)
	Second-order medium spatial frequency sensitivity	0.385	0.003	1.469 (1.136, 1.900)
	Second-order high spatial frequency sensitivity	0.390	0.002	1.476 (1.150, 1.895)
Model III	First-order low spatial frequency sensitivity	0.081	0.804	1.085 (0.570, 2.066)
	First-order medium spatial frequency sensitivity	0.493	0.158	1.637 (0.826, 3.245)
	First-order high spatial frequency sensitivity	0.260	0.366	1.297 (0.738, 2.279)
	Second-order low spatial frequency sensitivity	0.442	0.009	1.556 (1.116, 2.168)
	Second-order medium spatial frequency sensitivity	0.328	0.015	1.388 (1.066, 1.806)
	Second-order high spatial frequency sensitivity	0.389	0.003	1.476 (1.139, 1.912)

Model I, No adjustments were made; Model II, adjusts for age and gender; Model III, adjusts for age, gender, glycated hemoglobin, diabetes duration, BMI, and hypertension. Odds Ratio (OR); 95% confidence interval (95% CI); regression coefficient (B).

### 3.4 Exploring factors influencing first- and second-order high spatial frequency sensitivity

The above analysis results show that the first-and second-order high spatial frequency sensitivity was significantly associated with increased risks of DR. Therefore, this section further explores factors influencing first-and second-order high spatial frequency sensitivity. The Pearson correlation analysis results reveal a significant positive correlation between first-order high spatial frequency sensitivity and glycated hemoglobin(*r* = 0.105, *p* = 0.015), and age (*r* = 0.112, *p* = 0.009). Besides, there is a significant positive correlation between second-order high spatial frequency sensitivity and glycated hemoglobin (*r* = 0.119, *p* = 0.005) (Table 4).

**Table 4:** Exploring factors influencing first- and second-order high spatial frequency sensitivity.

	Glycated hemoglobin *r* (*p*)	Age *r* (*p*)	BMI *r* (*p*)	Diabetes duration *r* (*p*)
First-order high spatial frequency sensitivity	0.105 (0.015)	0.112 (0.009)	−0.008 (0.859)	0.004 (0.935)
Second-order high spatial frequency sensitivity	0.119 (0.005)	0.04 (0.352)	−0.006 (0.884)	−0.002 (0.971)

### 3.5 The dose–response relationship between first- and second-order high spatial frequency sensitivity and glycated hemoglobin

The above research results show that there is a positive correlation between first-and second-order high spatial frequency sensitivity and glycated hemoglobin. To further study the dose–response relationship between them, this study adopts a restricted cubic spline analysis combining spline functions and linear regression.

**Figure 2:**
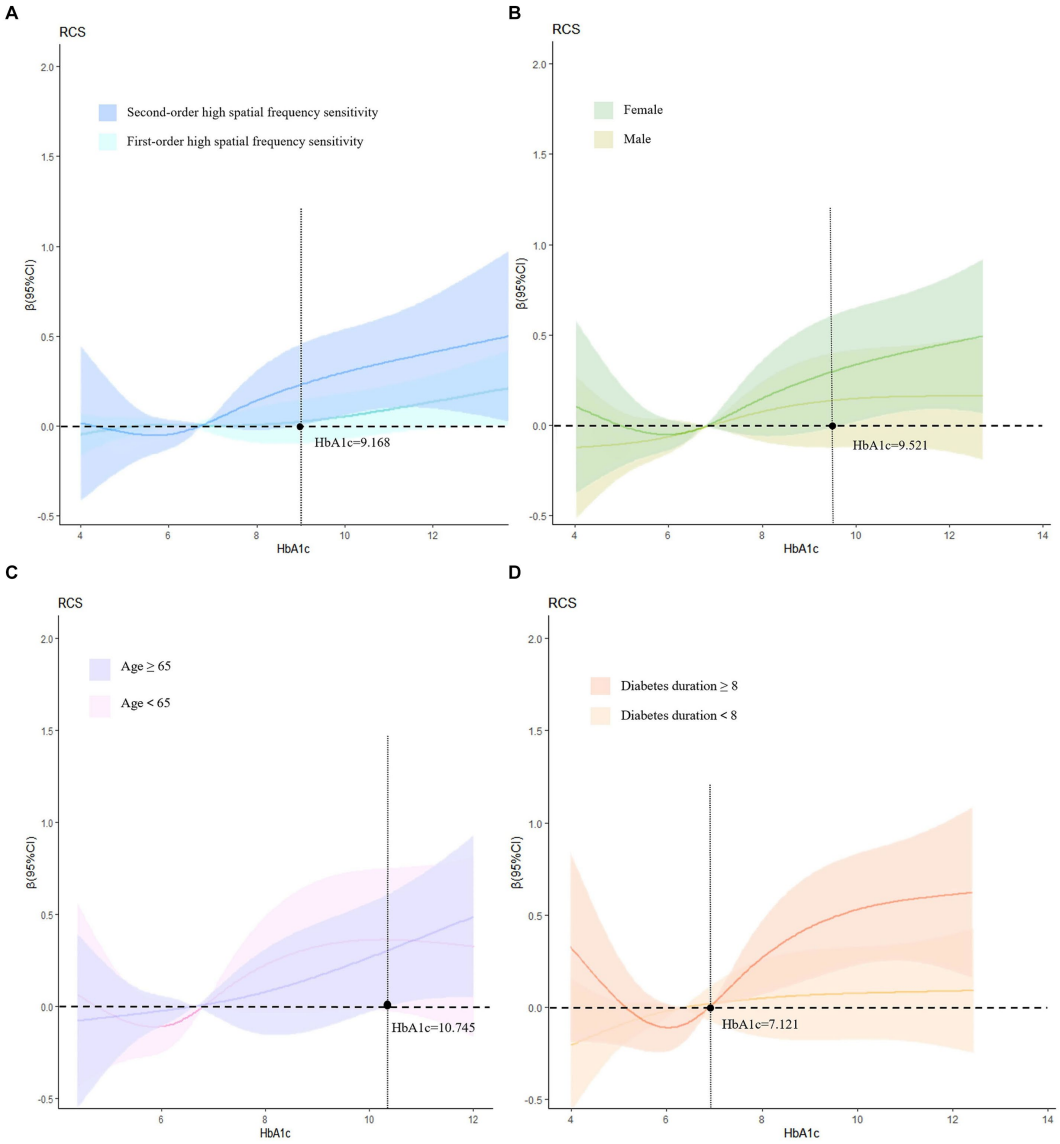
Dose–response relationship and subgroup analysis. **(A)** Adjusts for age, gender, diabetes duration, hypertension, and BMI; **(B)** adjusts for age, diabetes duration, BMI, and hypertension; **(C)** adjusts for gender, diabetes duration, BMI, and hypertension; **(D)** adjusts for age, gender, BMI, and hypertension. HbA1c, glycosylated hemoglobin.

The results indicated that there was no statistically significant dose–response relationship between first-order high spatial frequency sensitivity and glycated hemoglobin levels, when glycated hemoglobin exceeded 9.168%, there was an increasing trend in second-order high spatial frequency sensitivity (Figure 2A). Therefore, subgroup analysis was conducted on the dose–response relationship of second-order high spatial frequency sensitivity. In the subgroup analysis after adjusting for relevant factors, when the glycated hemoglobin in women exceeds 9.521% (Figure 2B), the glycated hemoglobin in the age ≥ 65 groups exceeds 10.745% (Figure 2C), and the glycated hemoglobin in diabetes duration≥8 groups exceeded 7.121% (Figure 2D), the second-order high spatial frequency sensitivity shows an upward trend (Figure 2).

## 4 Discussion

In the past, most previous studies on the pathogenesis of DR have primarily focused on microangiopathy in the retina. However, there is now a growing understanding of the complexity of the neurovascular unit in the retina. Numerous studies have demonstrated that neuropathy is present in the early stages of DR, with nerve damage preceding the microvascular abnormalities of DR. This damage progresses before the first clinical signs of DR appear. The primary manifestations of this damage include reduced contrast sensitivity at low spatial frequencies (Safi et al., 2017), abnormal results in color vision tests (Gella et al., 2015; Tan et al., 2017), and prolonged implicit time in multifocal electroretinogram recordings (Bearse et al., 2004; Laron et al., 2012). A study on the analysis of functional brain networks found that brain areas related to visual function (including primary visual cortex, secondary visual cortex, and visual information processing areas) showed reduced clustering coefficients and eigenpath lengths as well as increased degree distributions in patients with DR, which suggests that visual function is weaker in patients with DR (Dai et al., 2017). Another study, using resting-state MRI on 21 patients with DR, found that the strength of functional brain network connectivity was reduced in patients with DR compared to healthy subjects in the control group and that the reduction involved the visually related cortex (Li et al., 2019). All of the above studies suggest that diabetic patients can have early central visual impairment before the onset of retinopathy and that patients with DR may have a decline in visual cognitive function before the onset of DR. The results of our study indicate a reduction in both first- and second-order high spatial frequency sensitivity perception in diabetic patients with DR, which supports this conclusion. Furthermore, our study has revealed that among type 2 diabetes patients already diagnosed with DR, all three spatial frequencies of second-order spatial frequency can contribute to the incidence of moderate to advanced DR, while the effect of first-order spatial frequency is not significant. The analysis of visual motion information involves multiple cortical regions from V1 (primary visual cortex), V2, V3, MT, MST, IPL, and SPL, and the formation sites for second-order high spatial frequency motion perception are also in the higher cortical regions such as V2 and V3 (Muckli et al., 2002). Therefore, it is speculated that the probability of type 2 diabetes patients developing mid-to-late stage DR increases when higher visual cortical areas such as V2 and V3 are damaged.

In further analysis of the factors influencing the visual motion sensitivity test, it was found that glycated hemoglobin is a significant factor affecting first-order and second-order high spatial frequency motion. This may be due to the fact that glycated hemoglobin, which is used as a biomarker for detecting and monitoring diabetes mellitus, can have a negative impact on the central nervous system and, consequently, the visual cortex, resulting in impaired visual cognitive abilities. Therefore, it is important to consider the potential effects of glycated hemoglobin when interpreting results from visual motion sensitivity tests. Findings from the Diabetes Control and Complications Trial (DCCT) and the Epidemiology of Diabetes Interventions and Complications (EDIC) have shown that prolonged hyperglycemia, as well as microvascular lesions in the eyes and kidneys, have important biomedical correlates with cognitive decline (Jacobson et al., 2011). Wang et al. (2012) studied the effects of untreated hyperglycemia on brain metabolism from the onset to the chronic stage, where prolonged uncontrolled hyperglycemia can be detrimental to the central nervous system.

Further stratified analyses indicate that the dose–response relationship between glycated hemoglobin and second-order high spatial frequency motion perception varies across genders, ages, and diabetes duration. After conducting a meta-regression analysis on the average age of diabetic patients, the duration of diabetes, and glycated hemoglobin levels, it was found that the duration of diabetes is negatively correlated with the volume of gray matter in the right cerebral cortex, the average age of patients and glycated hemoglobin levels do not exhibit a linear relationship with changes in gray matter volume (Liu et al., 2017). Therefore, we infer that there may be some relationship between the duration of diabetes and central nervous system damage. Furthermore, a study performed the Farnsworth-Munsell 100 color phase test on 872 diabetic patients without DR and showed that 67 patients who were trichromatic abnormal females, older, and had a longer history of diabetes were more susceptible to the effects of color vision impairment (Shoji et al., 2011). Color vision deficits are associated with other functional and structural retinal abnormalities and are detected before any clinical signs of DR appear (Tan et al., 2017). Therefore, the older the patient is and the longer he or she has had diabetes, the more important it is for the patient to control glycated hemoglobin and focus on changes in visual cognitive function thereby preventing DR.

This study has several limitations. Firstly, the visual motion sensitivity test utilized in this study may not be applicable to all diabetic patients. For instance, patients with impaired vision, advanced age, or severe disease may not be able to effectively participate in the test. Secondly, the patients included in this study were predominantly middle-aged and older adults, and additional research is necessary to ascertain whether the alterations in visual motion sensitivity observed in diabetic patients of other age groups are congruent with our findings. Finally, the sample size in this study was relatively small, and future studies should aim to increase the sample size to enhance statistical reliability.

In conclusion, this study investigates the relationship between visual motion sensitivity and the presence of DR in patients with type 2 diabetes, as well as the factors that influence visual motion sensitivity. The results indicate that visual motion sensitivity has a significant impact on the prevalence of DR, particularly in relation to second-order spatial frequency. Among the three different levels of spatial frequency sensitivity, high spatial frequency sensitivity has the greatest effect on the incidence of DR. Furthermore, glycated hemoglobin is an important factor that affects first- and second-order high spatial frequency sensitivity. These findings could provide valuable insights for early intervention, prognosis, and improved quality of life for patients with type 2 diabetes and DR. Future research will continue to explore this area in greater depth.

## Data availability statement

The raw data supporting the conclusions of this article will be made available by the authors, without undue reservation.

## Ethics statement

The studies involving humans were approved by Ethics Committee of Guizhou Center for Disease Control and Prevention (S2023-12). The studies were conducted in accordance with the local legislation and institutional requirements. The participants provided their written informed consent to participate in this study.

## Author contributions

TZ: Data curation, Formal analysis, Investigation, Methodology, Writing – original draft, Writing – review & editing. HY: Data curation, Formal analysis, Investigation, Methodology, Writing – original draft, Writing – review & editing. HW: Investigation, Visualization, Writing – review & editing. FoZ: Investigation, Visualization, Writing – review & editing. QP: Investigation, Project administration, Writing – review & editing. QZ: Investigation, Project administration, Writing – review & editing. FuZ: Investigation, Project administration, Writing – review & editing. QA: Writing – review & editing, Investigation, Project administration. TL: Funding acquisition, Methodology, Supervision, Writing – review & editing. YH: Funding acquisition, Methodology, Supervision, Writing – review & editing. YZ: Funding acquisition, Methodology, Supervision, Writing – review & editing.
